# Letting the data speak for themselves: a fully Bayesian approach to transcriptome assembly

**DOI:** 10.1186/s13059-014-0498-8

**Published:** 2014-10-31

**Authors:** Marcel H Schulz

**Affiliations:** Excellence Cluster for Multimodal Computing and Interaction, Saarland University, Saarbrücken, Saarland 66123 Germany; Department for Computational Biology and Applied Computing, Max Planck Institute for Informatics, Saarbrücken, Saarland 66123 Germany

## Abstract

A novel method for transcriptome assembly, Bayesembler, provides greater accuracy without sacrifice of computational speed, and particular advantages for alternative transcripts expressed at low levels.

## Challenges of transcriptome assembly from short read data

RNA-seq has become the *de facto* standard for the analysis of genome-wide gene expression. Nowadays, RNA-seq generates hundreds of millions of short read-fragments from expressed RNAs and enables the detection of thousands of expressed transcripts in just one sequencing run. A fundamental unsolved problem, however, is the problem of transcriptome assembly: collating short read sequences into the full-length transcripts from which they were derived. A new method from Anders Krogh and colleagues, published in this issue of *Genome Biology*, provides a novel approach to this solve this task [1].

The problem of transcriptome assembly from short read data is a hard one for a number of reasons. First, in higher eukaryotic organisms, each gene often produces a large number of different alternative transcripts, and many transcripts will share the majority of exons.

Second, owing to the short length of read fragments - for example, when using Illumina technology - alternative splicing events in a gene may be further apart than read or fragment length. This leads to a disambiguation problem, whereby the read data alone might not contain enough information to distinguish between different sets of transcripts that could give rise to the same set of alternative exon combinations.

Third, in RNA-seq experiments, the number of reads for each transcript correlates with the expression level of the transcripts. Therefore, transcripts that are expressed at low levels are hard to assemble; for example, this applies to minor splice variants and many long noncoding RNAs.

Finally, RNA-seq protocols have been shown to contain many biases that affect read coverage along the transcripts - for example, amplification bias or biases due to read mapping, which complicates the modeling of read distributions.

Methods for reference-based transcriptome assembly start with the alignment of reads to the genome and the construction of splicing graphs that define possible exon regions and pairwise connections between them. The read coverage on exons and exon connections is used to prioritize possible transcripts that can be generated from the splicing graph. Even with perfect data, the genes from which many transcripts are simultaneously expressed cannot be correctly assembled [2,3] as the number of possible transcripts for a splicing graph grows rapidly with the number of exons. Luckily, often there is only one major isoform per gene expressed for a given condition, meaning that these hard cases remain the exception rather than the rule.

## Current methods for transcriptome assembly

Over the past few years, many different approaches have been suggested to solve the transcriptome assembly problem from splicing graphs. In one approach to transcriptome assembly, the popular Cufflinks assembler constructs a graph that models conflicts between read pairs and finds the minimal transcript set that fully explains all observed read pairs [4]. The expression levels for all transcripts are estimated using a statistical method. Although elegant and sufficiently fast, the disadvantage of Cufflinks, and similar earlier approaches, is that the transcriptome assembly task is decoupled from the task of inferring the transcript expression levels. However, both tasks are interdependent, and the hope is that solving both tasks simultaneously would help to resolve the otherwise ambiguous cases where alternative exon regions are further apart than read or fragment length, as mentioned above. However, doing so makes the problem more complex as, theoretically, the expression of all possible transcripts, and combinations thereof, needs to be considered by the method.

The common solution is to make the assumption that few transcripts per gene are expressed. In practice, that means that the solution sought is parsimonious in terms of the number of transcripts while explaining most of the mapped reads, which is often at the expense of providing accurate information about transcripts that are expressed at low levels.

Different approaches have been proposed, including statistical methods that model read distributions along transcripts, possibly accounting for RNA-seq biases. These methods minimize the error between the expression of assembled transcripts and observed read abundances by using optimization methods [3,5-7]. Another group of methods model the expression of transcripts in the splicing graph as flow through a network, which has been shown to lead to efficient algorithms [8,9].

Other than differences in the underlying assumptions of read coverage distributions and the incorporation of RNA-seq biases, these methods differ in the way they handle the exploding number of possible transcripts. Exhaustively exploring all possible transcript combinations, given the constraint of enforcing a minimal number of expressed transcripts, is computationally intractable for genes with many exons. Therefore, methods either use stronger constraints that lead to a reduced search space that can be explored efficiently [5,7,9] or heuristics are employed that limit the number of considered transcript combinations [3] to improve the runtime in practice. Although successful, a trade-off is made in order to tackle the complexity, and it can be expected that these modeling approaches perform suboptimally for some genes.

## A Bayesian approach to transcriptome assembly

In this issue of *Genome Biology*, Maretty, Sibbesen and Krogh, researchers from the University of Copenhagen, have introduced a new approach to transcriptome assembly [1]. The authors combined a graphical model that describes the RNA sequencing process, which had been suggested earlier, with fully Bayesian parameter inference and a Gibbs sampling strategy. Gibbs sampling is a strategy to explore, through random sampling, a large space of possible parameter configurations.

Instead of removing transcripts that are expressed at low levels but that are possibly correct, before final optimization, the ‘Bayesembler’ lets the data speak for themselves. If a transcript combination is unlikely to be generated by the data, the Gibbs sampler is unlikely to report this combination in a sampling round. However, the true set of transcripts and closely related solutions will have a high probability and will be returned in many sampling rounds. After many thousands of sampling rounds, the most likely transcript set can be deduced by averaging over all samples.

In their paper, the authors benchmark Bayesembler against other assemblers that are currently used in practice. They compare the results on simulated and real RNA-seq data sets for human and mouse. They show that the Bayesembler has the following properties: first, it assembles more transcripts with higher precision; second, it estimates transcript abundances more accurately; third, it introduces fewer errors in the assembly; and finally it shows the highest reproducibility among replicate samples in comparison with the other methods tested.

Importantly these advantages do not come at the cost of increased runtime, which can be a problem with sampling-based approaches. Bayesembler can use the multiple cores of a computer to speed up computations and is reported to run faster than the widely used Cufflinks assembler.

There are also other interesting advantages of the new approach. First, many transcriptome assembly methods involve parameters that would be worthwhile to adjust for a new data set to improve the assembly result. This requires the users’ expertise, which means that less-experienced users might get suboptimal performance on their data set. However, the Bayesian treatment in Bayesembler avoids the need to tune parameters for a new data set, which should allow easy integration into existing bioinformatics workflows.

Also, previous methods produce a single final set of assembled transcripts, despite the fact that there might be several equally good solutions. In contrast, the Bayesembler directly provides confidence estimates for assembled isoforms and their expression levels by sampling also suboptimal solutions. These confidence estimates not only allow the prioritization of potentially novel transcripts for validation studies but they could also be used to carry over the uncertainty of the assembly process to other downstream analyses, such as differential transcript expression computation.

Furthermore, many of the ideas in the Bayesembler can be extended to other variants of the problem, such as reference-assisted or complete *de novo* transcriptome assembly. Here again, confidence estimates for assemblies should prove useful.

## Concluding remarks

Finally, community-driven competitions, similar to the study published last year by the RGASP consortium [10], or other carefully designed benchmarking studies, will be necessary to fully understand how far we are from solving the transcriptome assembly problem using methods such as the Bayesembler and other recent approaches.
